# Ferroptosis: a novel mechanism of cell death in ophthalmic conditions

**DOI:** 10.3389/fimmu.2024.1440309

**Published:** 2024-06-27

**Authors:** Yaqi Yang, Yumeng Lin, Zhongyu Han, Bo Wang, Wei Zheng, Lijuan Wei

**Affiliations:** ^1^ College of Chinese Medicine, Changchun University of Chinese Medicine, Changchun, Jilin, China; ^2^ Naniing Tongren Hospital, School of Medicine, Southeast University, Nanjing, China; ^3^ Ophthalmology Department, Affiliated Hospital of Changchun University of Traditional Chinese Medicine, Changchun, Jilin, China

**Keywords:** ferroptosis, metabolic pathway, eye diseases, therapy, corneal injury

## Abstract

Ferroptosis, a new type of programmed cell death proposed in recent years, is characterized mainly by reactive oxygen species and iron-mediated lipid peroxidation and differs from programmed cell death, such as apoptosis, necrosis, and autophagy. Ferroptosis is associated with a variety of physiological and pathophysiological processes. Recent studies have shown that ferroptosis can aggravate or reduce the occurrence and development of diseases by targeting metabolic pathways and signaling pathways in tumors, ischemic organ damage, and other degenerative diseases related to lipid peroxidation. Increasing evidence suggests that ferroptosis is closely linked to the onset and progression of various ophthalmic conditions, including corneal injury, glaucoma, age-related macular degeneration, diabetic retinopathy, retinal detachment, and retinoblastoma. Our review of the current research on ferroptosis in ophthalmic diseases reveals significant advancements in our understanding of the pathogenesis, aetiology, and treatment of these conditions.

## 1 Introduction

The pathology of ophthalmic diseases involves various mechanisms of cell death, including apoptosis, necrosis, and autophagy (1, 2). It is specifically regulated by iron levels and the antioxidant enzyme glutathione peroxidase 4 (GPX4), unlike apoptosis’s caspase-dependent mechanism, necrosis’s inflammatory cell rupture, and autophagy’s dual role in both cell survival and death (3). Each form of regulated cell death (RCD) exhibits distinct morphological features and is controlled by unique biochemical, genetic, and functional processes. Studies have shown that pharmaceutical agents modulate apoptosis, necrosis, and autophagy within ocular cells by targeting pathways such as inflammation, oxidative stress, and endoplasmic reticulum stress, leading to positive therapeutic effects (4).

The key membrane protein xCT related to ferroptosis was discovered as early as the 1980s (5). Subsequently, over the course of more than 20 years, the classic inducers of ferroptosis, erastin, which can kill RAS-mutated BeJLR cells, were also discovered in 2003 (6). However, it was not until 2012 that researchers such as Dixon defined this type of cell death as ferroptosis. Dixon and colleagues identified a form of programmed cell death (PCD) reliant on iron metabolism, particularly in cancer cells (7). The term “ferroptosis” was coined to describe a new form of non-apoptotic cell death activated by the small molecule erastin, which was found to induce cell death through iron-dependent lipid peroxidation.

**Figure 1:**
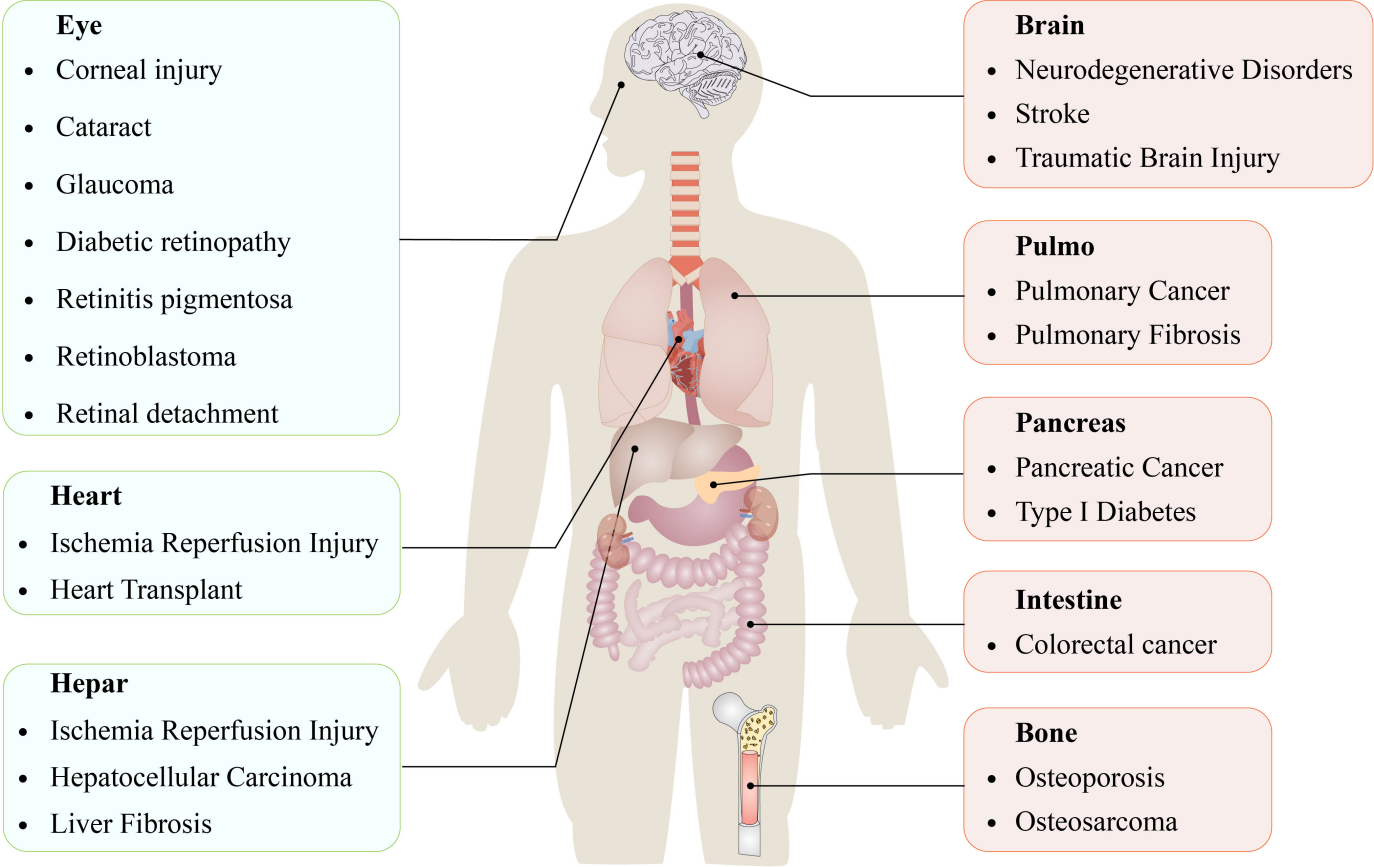
Ferroptosis is involved in the occurrence and development of various diseases in organs and tissues.

This phenomenon is observed not only in mammals but also in plants, protozoa, and fungi, including *Magnaporthe oryzae* and *Caenorhabditis elegans* (8, 9). Emerging evidence suggests that ferroptosis significantly regulates the progression of numerous systemic diseases (**Figure 1**) (10–17).

It is widely believed that ferroptosis primarily results from an overload of intracellular reactive oxygen species (ROS) dependent on iron, combined with a reduction in the detoxifying activity of GPX4, leading to an imbalance in ROS production and clearance (7). When the cell’s antioxidant defenses are overwhelmed and cannot neutralize the excess ROS, ferroptosis is triggered within the cell, with the subsequent peroxidation reactions being pivotal to its onset (18). Consequently, numerous molecules and signaling pathways involved in iron metabolism and peroxidation reactions are pivotal in the modulation of ferroptosis.

This review delves into the latest developments and future prospects in the research of ferroptosis in corneal injury, glaucoma, age-related macular degeneration, diabetic retinopathy, retinal detachment, and retinoblastoma. This study investigates the metabolic interplay between ferroptosis and ophthalmic pathology, aiming to establish a foundation for further exploration of the mechanisms and preventive strategies of ferroptosis in ophthalmic diseases.

## 2 Characteristics of ferroptosis

Iron ions play a pivotal role in metabolism in the ocular region, serving as essential micronutrients for a wide array of biological activities (19). They contribute significantly to the light conversion process in photoreceptor cells in the retina, participate in the electron transport chain within mitochondria, and are crucial for the activity of enzymes such as cytochrome oxidase (20). Excess iron ions produce ROS through the Fenton reaction, and iron overload and ROS accumulation are involved in the pathophysiological mechanisms of ferroptosis (21). Ferroptosis is a form of programmed cell death triggered by excessive iron accumulation and lipid peroxidation-induced damage. Signaling pathways and molecules involved in the regulatory mechanism of ferroptosis include iron metabolism, cysteine metabolism, GPX4 inactivation, polyunsaturated fatty acid (PUFA) synthesis, nuclear factor E2-related factor 2 (Nrf2), p53, heat shock proteins (HSPs), iron-regulated inhibitor-1 (FSP1), AMP-activated protein kinase (AMPK) activation, and nicotinamide adenine dinucleotide phosphate (NADPH), etc. We will classify and introduce ferroptosis-related molecules from signaling, execution, regulation and other functions.

### 2.1 Signaling

#### 2.1.1 Iron metabolism

**Figure 2:**
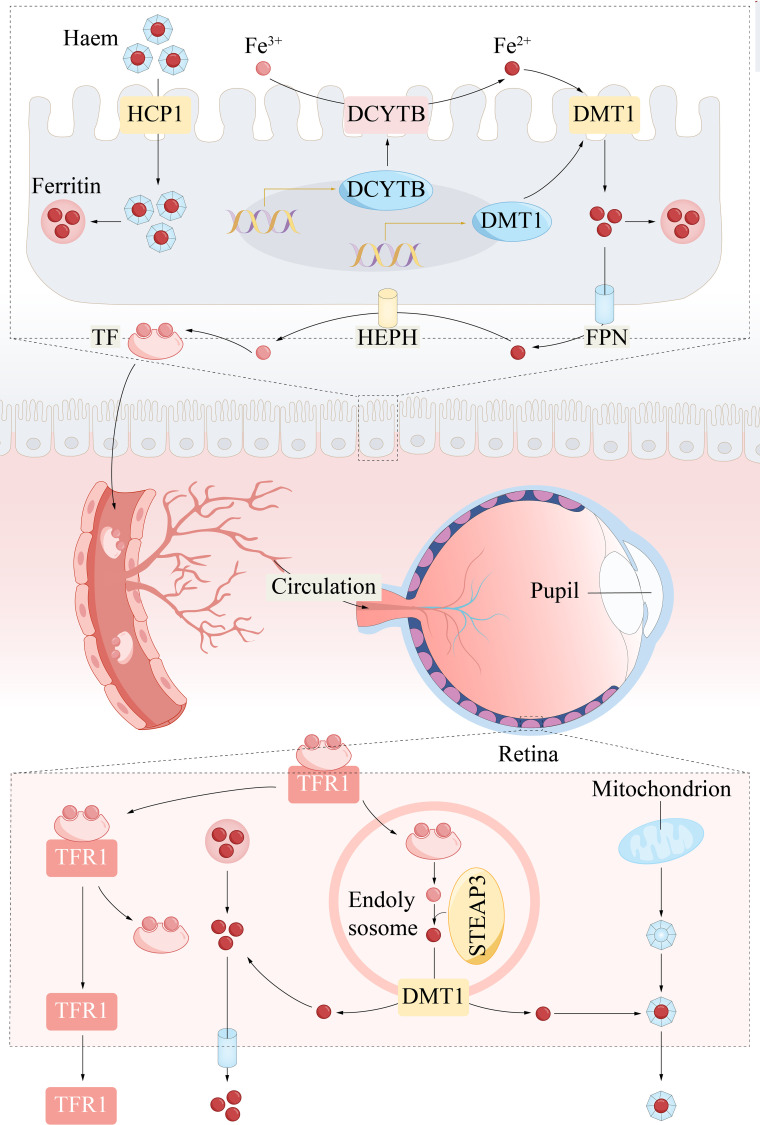
Systemic circulation of iron and iron metabolism. Iron ions are absorbed from the gut and reach the eyeball as blood circulates throughout the body. Specifically, Fe^3+^ is reduced to Fe^2+^ by DCYTB in the intestinal lumen. DMT1 then transports Fe^2+^ into enterocytes. HCP1 also incorporated heme iron into duodenal enterocytes. HIF2α (not shown, in the nucleus) binds to hypoxia response elements (HRE) in the regulatory regions of the DMT1 and DCYTB promoters and is therefore post-transcriptionally regulated. Intracellular iron storage is controlled by ferritin, whereas Fe^2+^ is exported into the blood via FPN and simultaneously oxidized to Fe^3+^ by hephaestin. Plasma transferrin captures and distributes Fe^3+^ to the retina. Cells take up iron via TFR1 -mediated endocytosis of holotransferrin. In retinal cells, DMT1 transports Fe^2+^ into the cytoplasm. Fe^2+^ can shuttle to mitochondria for heme biosynthesis. DCYTB, duodenal cytochrome B; DMT1, divalent metal transporter 1; FPN, ferroportin; HEPH, hephaestin; STEAP3, six transmembrane epithelial antigen of the prostate 3; TF, transferrin; TFR1, transferrin receptor 1.

Iron metabolism is crucial in ferroptosis, influencing the accumulation of lipid peroxides (22). Iron uptake, iron transport across cell membranes, and iron storage profoundly influence the regulation of ferroptosis (23). Organisms carefully maintain a delicate balance to maintain iron homeostasis. The acquisition of iron through transferrin receptor 1 (TFR1) is crucial for ferroptosis; this receptor facilitates the transport of ferric ions (Fe^3+^) to endosomes, where they are converted to ferrous ions (Fe^2+^) (**Figure 2**). Divalent metal transporter 1 (DMT1) releases Fe^2+^ from endosomes into the labile intracellular iron pool. Excess iron is then sequestered within the cytoplasm in the form of ferritin light chain (FTL) and ferritin heavy chain 1 (FTH1) (24). Disruptions in the normal expression or function of these proteins related to iron metabolism can lead to an increase in intracellular iron levels, resulting from metabolic dysregulation (25). Under conditions of iron surplus, ferroportin 1 (FPN1) is notably expressed on the brush border membrane of renal proximal tubule cells, indicating its potential role in the excretion of excess iron into the urinary space (26). Hepcidin, which is predominantly produced in the liver, plays a crucial role in governing the release of iron by FPN1 in macrophages (27).

Iron plays a crucial role in the visual process, particularly in phototransduction, where it acts as a cofactor for key enzymes involved in the regeneration of rhodopsin, converting light signals into neural signals. Additionally, iron is involved in maintaining the antioxidant balance of the retina, helping to eliminate free radicals and protect the retina from oxidative damage (28). However, disruptions in iron metabolism can lead to iron deposition and lipid peroxidation, which may have a negative impact in age-related macular degeneration (29). Therefore, the balance of iron ions is vital for retinal health, and excessive light exposure may cause iron metabolic disorders and harmful oxidative stress, damaging retinal cells.

### 2.2 Execution

#### 2.2.1 GPX4 inactivation

**Figure 3:**
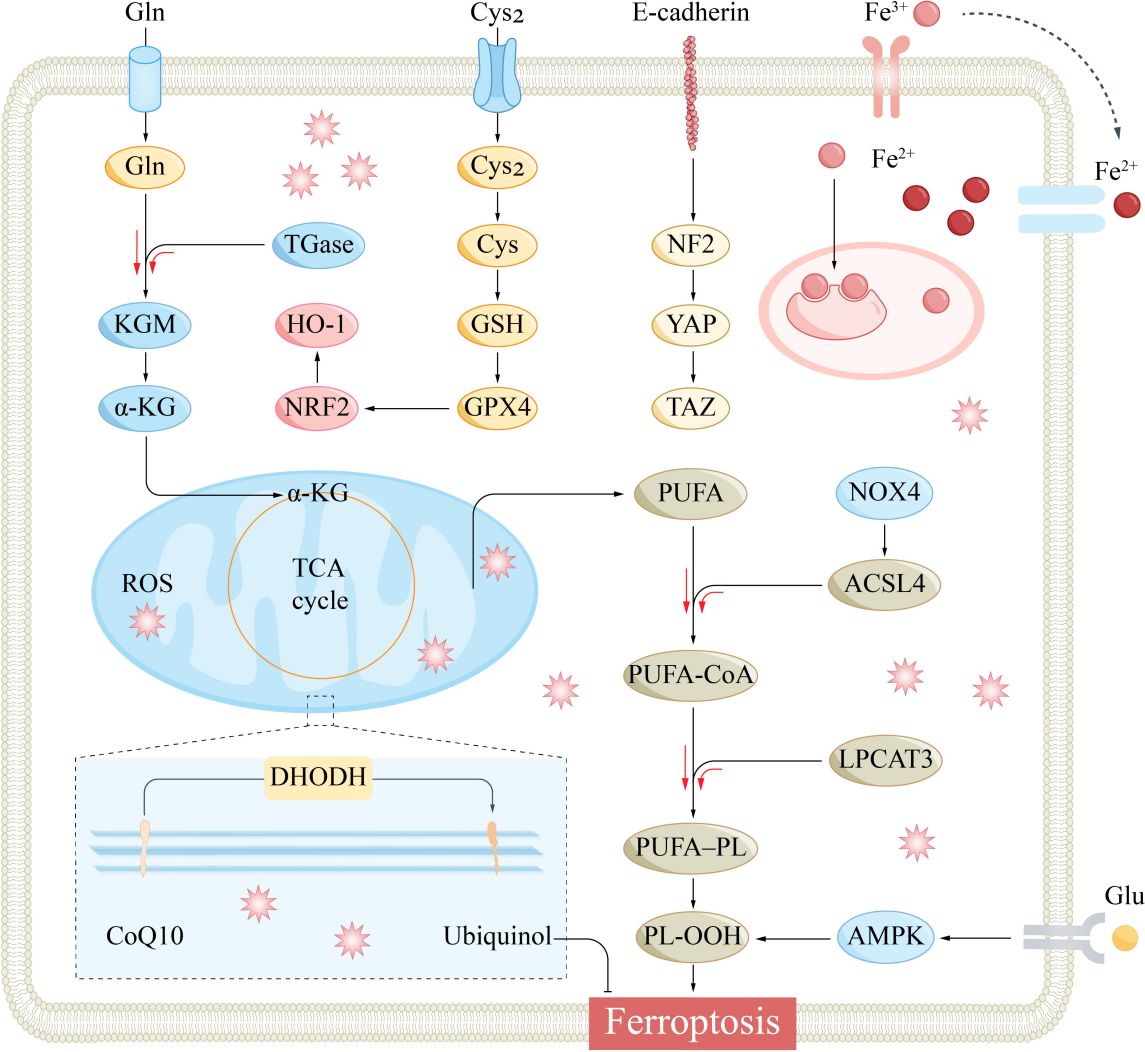
The main signaling pathways that regulate ferroptosis. Specifically, Cysteine metabolism, Iron metabolism, GPX4 inactivation, PUFA synthesis, Hippo pathway, AMPK, and NRF2 are involved in the regulatory mechanisms of ferroptosis. α-KG, α-ketoglutarate; ACSL4, acyl-CoA synthetase long-chain family member 4; AMPK, AMP-activated protein kinase; CoQ10, coenzyme Q10; Cys2, cystine; DHODH, dihydroorotate dehydrogenase; Gln, glutamine; Glu, glutamate; GPX4, glutathione peroxidase 4; GSH, glutathione; HO-1, heme oxygenase-1; KGM, konjac glucomannan; LPCAT3, lysophosphatidylcholine acyltransferase 3; NF2, Neurofibromin 2; NOX4, NADPH oxidase 4; Nrf2, nuclear factor erythroid 2-related factor 2; PL-OOH, phospholipid hydroperoxide; PUFA, polyunsaturated fatty acid; PUFA-PL, phospholipid containing polyunsaturated fatty acid chain; ROS, reactive oxygen species; TCA, tricarboxylic acid cycle; YAP, yes-associated protein.

The glutathione peroxidase (GPX) family comprises various isoforms, including GPX1 through GPX8, with GPX4 playing a particularly crucial role in the context of ferroptosis (**Figure 3**) (30). GPX4, a selenoprotein in mammals, is essential for the repair of lipid peroxidation within cells (31). It facilitates the conversion of the reduced form of glutathione (GSH) to its oxidized counterpart (GSSG) and transforms toxic lipid hydroperoxides (L-OOH) into nontoxic lipid alcohols (L-OH), thereby catalysing the breakdown of hydrogen peroxide (H_2_O_2_). This process is vital for safeguarding the cell membrane’s structure and function against oxidative stress and damage (32).

GPX4 plays a crucial role in protecting retinal cells from oxidative stress and ferroptosis damage. It catalyzes the reduction of lipid peroxides by GSH, thereby protecting the cell membrane from oxidative damage, which is essential for maintaining retinal health (33). In ophthalmic diseases such as diabetic retinopathy, the downregulation of GPX4 may lead to increased oxidative stress, subsequently affecting the function and survival of retinal photoreceptor cells (34).

Agents that induce ferroptosis, such as RAS-selective lethal 3 (RSL3) and erastin, lead to an increase in lipid ROS levels (35). While erastin impacts GSH levels, RSL3 does not substantially alter GSH levels during ferroptosis (36). Subsequent investigations revealed that RSL3 specifically targets the GPX4 molecule, inhibiting its activity through covalent binding and resulting in the accumulation of lipid peroxides (37). Yang et al. noted that cells with diminished GPX4 expression are more susceptible to ferroptosis, whereas those with elevated GPX4 levels exhibit increased resistance (38). Consequently, GPX4 serves as a significant regulator of ferroptosis, and modulating its expression can either enhance or suppress the occurrence of ferroptosis (16).

#### 2.2.2 PUFA synthesis

PUFAs play a significant physiological role in the ophthalmic environment, particularly in terms of retinal health and visual function. PUFAs are crucial components of the cell membrane, essential for maintaining the fluidity and integrity of the retinal cell membrane (39). They are also involved in regulating lipid metabolism in the retina, playing an important role in promoting growth and development, immune modulation, and antioxidation.

The molecular structure of PUFAs features vulnerable carbon−carbon double bonds, making them susceptible to a process known as lipid peroxidation, a key event in ferroptosis (40). Two pivotal enzymes in the lipid remodeling process are acyl-CoA synthase long-chain 4 (ACSL4) and lysophosphatidylcholine acyltransferase 3 (LPCAT3), which are crucial for the production of PUFAs during ferroptosis (**Figure 3**) (41). The deletion or silencing of these genes can curtail the synthesis of PUFAs, thereby impeding the progression of ferroptosis (42, 43). Conversely, an abundance of arachidonic acid and other PUFAs can exacerbate the ferroptosis response when triggered by an inducer, as it accelerates the formation of peroxide lipids within the cell (40). This result highlights the significance of PUFAs as targets for plasma membrane peroxidation during ferroptosis.

Additionally, the enzyme lipoxygenase (LOX) facilitates the peroxidation of PUFAs, and decreasing LOX expression has been shown to mitigate ferroptosis, particularly that induced by erastin (40, 42). Given that free PUFAs serve as substrates for lipid peroxidation, their concentration and cellular distribution can influence the extent of lipid peroxidation and, consequently, the intensity of ferroptosis (42). Understanding the dynamics of PUFA localization and concentration presents an alternative avenue for modulating ferroptosis.

Some PUFAs, such as docosahexaenoic acid (DHA) and eicosapentaenoic acid (EPA), play a crucial role in visual development and retinal function. DHA is the most abundant omega-3 fatty acid in retinal photoreceptors and is essential for maintaining the structure and function of photoreceptor cells (44). EPA helps to reduce inflammatory responses and decrease the risk of age-related macular degeneration and other eye diseases. In addition, omega-3 PUFAs may also mitigate oxidative damage to ocular tissues through antioxidant mechanisms, protecting the retina from light-induced damage (45).

Future research may explore the possibility of modulating enzymes involved in the synthesis of phospholipids containing PUFAs as a strategy to either initiate or prevent ferroptosis. This approach could reveal novel regulatory mechanisms and potential therapeutic targets within the complex interplay of lipid metabolism and cell death pathways.

### 2.3 Regulation

#### 2.3.1 Cysteine metabolism

Cysteine metabolism plays a pivotal role in ferroptosis, serving as a critical regulatory hub for the synthesis of GSH, the primary antioxidant defense against the peroxidation of lipids within the cell membrane. The cystine/glutamate antiporter System Xc- is a complex composed of two subunits, the light chain (SLC7A11) and the heavy chain (SLC3A2), which are linked by disulfide bonds (46). This transporter plays a vital role in cellular antioxidant defense mechanisms. It operates by taking up cystine while exporting glutamate in an equimolar exchange (47). Once internalized, cystine is converted to cysteine, which is a critical component in the assembly of GSH. GSH, under the influence of GPX4, facilitates the conversion of harmful lipid peroxides into harmless fatty alcohols, thereby acting as a pivotal cellular antioxidant (**Figure 3**) (48).

In this biochemical cascade, GSH acts as a reducing agent, with GPX4 being the central enzyme that catalyses the reduction of lipid peroxides and thereby suppresses ferroptosis (49). Nonetheless, the rate of GSH production is contingent upon the availability of its precursor, cysteine, making System Xc- a crucial regulatory element. Compounds such as erastin, which are known to induce ferroptosis, impede GSH synthesis by directly targeting System Xc- and curbing cysteine uptake. A subsequent deficiency in GSH can result in the accumulation of detrimental peroxides, leading to protein and cell membrane damage and ultimately triggering ferroptosis (7).

Cysteine metabolism plays a crucial role in the ophthalmic environment, particularly in maintaining the health and function of the retina. Cysteine is the rate-limiting precursor for the biosynthesis of GSH, which is one of the primary antioxidants within cells and essential for protecting retinal cells from oxidative stress and ferroptosis. In the retinal pigment epithelial cells and photoreceptor cells, GSH helps to neutralize light-induced ROS, which are continuous byproducts of the visual cycle (50). One of the hallmarks of ferroptosis is the accumulation of lipid peroxides, and GSH, in its reduced form, aids in maintaining the integrity of the cell membrane, preventing lipid peroxidation.

#### 2.3.2 Nrf2

The Nrf2 pathway is integral to the cellular response against ferroptosis, acting as a master regulator of antioxidant and detoxification processes. In a 2016 study, Sun and colleagues showed that the transcription factor Nrf2 can modulate ferroptosis through the P62-KEAP1-NRF2 signaling axis (51) (**Figure 3**). Upon activation, Nrf2 enhances the sequestration of iron and reduces its cellular uptake, thereby curtailing ROS generation and exerting an inhibitory effect on ferroptosis (52). Furthermore, Nrf2 suppresses ferroptosis by upregulating the expression of genes pivotal for the metabolism of iron and ROS, including quinone oxidoreductase 1 (NQO1) and haem oxygenase-1 (HO-1) (53). Additionally, stimulation of the p62-KEAP1-NRF2 cascade can lead to the upregulation of System Xc-, which in turn augments the transport of cystine and glutamate. This accelerated transport aids in the elimination of accumulated lipid peroxides (54), contributing to the overall antioxidant response and cellular defense against ferroptosis.

Nrf2 mitigates oxidative stress-induced damage to ocular tissues by activating antioxidant enzymes that eliminate harmful reactive oxygen species and free radicals. It suppresses the production of inflammatory mediators, reducing ocular inflammatory responses, while also promoting the repair of damaged cells and the regeneration of retinal tissue, maintaining the integrity of ocular structures (55). Nrf2 also regulates cellular metabolic pathways, affecting the synthesis and breakdown of fatty acids, influencing the progression of metabolic diseases, and providing protective effects on retinal neurons, reducing neurodegeneration, and preserving visual function (56). Furthermore, it inhibits pathological angiogenesis, safeguarding vision, and is involved in the regulation of ferroptosis, the clearance of damaged organelles and misfolded proteins, thereby maintaining the stability of the intracellular environment (57). These integrated functions underscore the essential role of Nrf2 in maintaining ocular health and visual function.

#### 2.3.3 P53

The tumor suppressor p53, which is activated by various stress signals, has been implicated in regulating ferroptosis (58). By repressing the SLC7A11 gene at the transcriptional level, p53 contributes to the onset of ferroptosis by hindering the uptake of cysteine, an essential amino acid for GSH synthesis, thereby promoting ferroptosis (54). The role of p53 in inducing ferroptosis is associated with a specific mutational mechanism involving the substitution of three lysine residues with arginine. These mutations created an acetylated mutant form of p53 known as p533KR, which significantly suppresses the expression of SLC7A11 without affecting other recognized p53 target genes, such as those linked to cell cycle regulation, apoptosis, or ageing (59).

System Xc- is a crucial transporter for cystine and glutamate, forming a heterodimeric complex with the subunits SLC7A11 and SLC3A2 (60). Consequently, p53 impedes cystine uptake by System Xc- through the downregulation of SLC7A11. This reduction in cystine uptake leads to a decrease in cystine-dependent glutathione peroxidase (GPX) activity, which weakens the cell’s antioxidant defenses (61). As a result, lipid ROS levels are increased, triggering ferroptosis within the cell (62). This regulatory pathway underscores the importance of p53 in modulating the cellular susceptibility to ferroptosis and provides a potential therapeutic target for interventions aimed at preventing or treating conditions associated with this form of cell death.

P53 plays a critical protective role in ophthalmic diseases by monitoring cellular DNA damage and stress signals. When retinal cells undergo photic damage, hypoxia, or metabolic stress, P53 is swiftly activated to induce cell cycle arrest, thereby allowing time for DNA repair (63). If the damage is too severe, P53 initiates the programmed cell death process to prevent the proliferation of damaged cells and potential tumor formation.

Moreover, P53 is involved in regulating cellular metabolism, influencing the progression of eye diseases such as diabetic retinopathy by affecting blood sugar balance and fatty acid oxidation (64). The activity of P53 is also linked with neuroprotection in age-related macular degeneration and glaucoma (65). In these conditions, it fosters the expression of stress-resistant molecules to shield retinal neurons from degeneration.

#### 2.3.4 HSPs

HSPs are critical in modulating the cellular response to ferroptosis, serving as a network of molecular chaperones that assist in the preservation of cellular integrity under oxidative stress. HSPs form an evolutionarily conserved family of molecular chaperones that fulfil a variety of immunological functions (66). These functions include mitigating cellular stress, exhibiting antioxidant activity, modulating immune responses, and facilitating antigen presentation. Through these functions, HSPs confer cellular resilience to various modes of cell death, including ferroptosis (67). One member of this family, heat shock protein B1 (HSPB1), also known as HSP25 or HSP27, safeguards the integrity of the actin cytoskeleton. This function is achieved by mitigating ferroptosis through a reduction in iron uptake, thereby curtailing subsequent oxidative damage (68).

HSPB1 also regulates the expression of TFR1 (69). Furthermore, the protein kinase C-mediated phosphorylation of HSPB1 results in the upregulation of FTL and FTH1 expression (68). This increase in ferritin expression reduces the concentration of intracellular ferrous ions and the production of lipid ROS (70).

The HSPB1 pathway, shown to counteract erastin-induced ferroptosis in cancers like cervical, prostate, and osteosarcoma, reduces iron and ROS levels (68, 71). Additionally, another member of the HSP family, heat shock protein A5 (HSPA5), also known as binding immunoglobulin protein (BIP) or 78 kDa glucose-regulated protein (GRP78), is localized to the endoplasmic reticulum. HSPA5 can bind to GPX4, thereby significantly bolstering the cell’s antioxidant defenses (72).

HSPs play the role of protectors in ophthalmic diseases, participating in the maintenance of cellular proteostasis, especially when faced with damaging conditions such as oxidative stress, inflammation, heat shock, ultraviolet radiation, and metabolic stress. HSPs like HSP27, HSP70, and HSP90 can prevent protein aggregation and cellular dysfunction by promoting the proper folding of damaged proteins, repairing, or clearing misfolded proteins. In retinal diseases, HSPs help protect photoreceptor cells from light damage and slow the progression of age-related macular degeneration (73). In glaucoma and neurodegenerative eye diseases, the neuroprotective role of HSPs is particularly important, as they can protect retinal ganglion cells from mechanical stress or disease-related neurotoxic damage (74, 75). This intricate interplay between HSPs and cellular antioxidant systems presents a promising avenue for therapeutic intervention in conditions where ferroptosis contributes to pathology.

#### 2.3.5 FSP1

A comprehensive genome-wide screening study revealed the role of FSP1 in regulating ferroptosis. Interestingly, the FSP1-CoQ10-NAD(P)H pathway operates as a significant intracellular antioxidant mechanism (76). This complements the traditional GSH-GPX4 pathway to collaboratively suppress phospholipid peroxidation and inhibit ferroptosis.

Specifically, FSP1 facilitates the reduction of CoQ10 to Ubiquinol using NAD(P)H (77). This reaction subsequently captures lipid peroxyl radicals, thereby halting the propagation of phospholipid peroxidation. In parallel, the GPX4 and glutathione systems aid in neutralizing phospholipid hydroperoxides and free radicals, further dampening peroxidative processes. In instances of GPX4 deficiency, the FSP1-CoQ10-NAD(P)H pathway steps up as the primary mechanism for combating ferroptosis.

In 2019, research conducted by Bersuker and colleagues indicated that the deletion of FSP1 resulted in tumor cells losing their resistance to the iron regulatory compound RSL3, concomitant with a marked increase in intracellular iron levels (78). FSP1, known for its myristoylation, contributes to the composition of numerous cellular membranes, and any modification of its myristoylated sites can impair its ferroptosis-inhibiting capabilities (79).

Mechanistically, FSP1 exerts its effects through the quinone oxidoreductase activity associated with NADH, converting ubiquinone to ubiquinol, which in turn inhibits lipid peroxidation and ferroptosis (80). Ubiquinol can directly neutralize lipid-derived free radicals, thereby halting the process of lipid autoxidation. Alternatively, it can indirectly cease lipid autoxidation through the regenerative oxidation of α-tocopherol free radicals (78).

FSP1 plays an integral role in the repair processes of the cornea and retina, facilitating the healing of damaged tissues by promoting cell adhesion and migration (81). Following a corneal injury, the deposition of FSP1 can provide a temporary substrate for epithelial cell regeneration, thereby expediting wound closure and repair.

Moreover, FSP1 significantly contributes to the functionality of RPE cells. It assists in maintaining the polarity and integrity of RPE cells, which are vital for the health and function of photoreceptor cells. In vascular diseases such as diabetic retinopathy, FSP1 may play a role in regulating the growth of abnormal blood vessels and scar formation (82). It influences the remodeling of the extracellular matrix and angiogenesis processes through interactions with growth factors and cell surface receptors. FSP1 may also have a role in age-related macular degeneration, affecting the formation of neovascularization and scar tissue beneath the retina (81).

This intricate regulatory network underscores the multifaceted role of FSP1 in modulating the cellular susceptibility to ferroptosis, providing novel insights into the complex interplay between cellular metabolism and iron homeostasis in the context of oxidative stress and cell death.

#### 2.3.6 AMPK

AMPK serves as a pivotal sensor within the cell, monitoring energy metabolism and orchestrating a variety of metabolic pathways to maintain energy homeostasis (83). The activation of AMPK has been implicated in both the promotion and suppression of ferroptosis (84). The seemingly contradictory effects of AMPK on ferroptosis may stem from the intricate interplay of environmental factors and the degree of AMPK phosphorylation at specific sites (85–87).

Moreover, AMPK is recognized for its role in governing mitochondrial stability, which is integral to cellular health and the regulation of cell death pathways (88). The activation of AMPK triggers the degradation of ferritin, an iron-storage protein, leading to increased ROS levels and the initiation of ferroptosis (89, 90). A study identified AMPK as an upstream modulator of ferroptosis, and the depletion of AMPK sensitizes cells to this form of death (91). Conversely, another study reported that AMPK becomes activated under glucose-deprived conditions and that the AMPK signaling cascade curbs lipogenesis, thereby reducing the propensity for ferroptosis (**Figure 3**) (92).

In addition, AMPK is also involved in the regulation of mitochondrial biogenesis and inflammatory responses, both of which are significant factors in the development of eye diseases. For instance, the activation of AMPK can promote the process of mitochondrial ferroptosis, assisting in the replacement of dysfunctional mitochondria, which is crucial for maintaining the health of ocular cells (93). At the same time, AMPK can also inhibit the mTORC1 pathway, a key regulatory factor for cell growth and proliferation, and its inhibitory effect helps to control the progression of eye diseases (94). Therefore, the regulatory role of AMPK has potential application value in ophthalmic treatment and can serve as a new strategy for treating eye diseases.

#### 2.3.7 NADPH

NADPH is a vital coenzyme in cellular redox homeostasis and plays a crucial role in ferroptosis by functioning as the primary reducing equivalent to counteract oxidative stress. It functions as a coenzyme for glutathione reductase, is integral to maintaining intracellular GSH levels (95). The influence of well-established ferroptosis initiators, including erastin, RSL3, and FIN56, has been explored across diverse cell lineages, notably those derived from human osteosarcoma (96). These studies consistently reported a significant decrease in the levels of intracellular NAD(H) and NADP(H), alongside the detection of ROS formation (97).

Current research shows NADPH donates electrons to cytochrome P450 via P450 oxidoreductase (POR), supplying them with electrons (98). This electron transfer may precipitate lipid peroxidation through several proposed mechanisms, either by facilitating the dehydrogenation of PUFAs or by impeding the reduction of Fe^3+^ to Fe^2+^ (99, 100).

These findings suggest that NADPH not only is central to the cellular redox balance but also plays a critical role in regulating ferroptosis, potentially by influencing iron metabolism and lipid peroxidation (101). Understanding the nuances of the role of NADPH in these processes could provide novel perspectives on the modulation of ferroptosis and its implications in various pathophysiological conditions.

Besides, NADPH is a key coenzyme for the synthesis of lipids and cholesterol within cells, which is crucial for the membrane structure and function of retinal cells. In ophthalmic diseases, the synthesis and utilization of NADPH may be affected, leading to dysfunction of retinal cells (102). Therefore, maintaining the levels and functionality of NADPH is essential for protecting vision and treating ophthalmic diseases (103). Supplementing NADPH or enhancing its synthetic pathways may help improve retinal health and delay the progression of ophthalmic diseases.

#### 2.3.8 GCH1

A laboratory investigation revealed that GCH1, an essential enzyme in the biosynthesis of folates, can prevent ferroptosis (104). This protective effect is believed to be mediated by its metabolic products, tetrahydrobiopterin (BH4) and dihydrobiopterin (BH2), which are crucial for cellular redox homeostasis and the maintenance of nitric oxide synthase function (105).

Complementary lipidomic profiling revealed that cells with elevated GCH1 levels contain two PUFA chains that seem to shield phosphatidylcholine phospholipids from peroxidative damage. These PUFA chains may contribute to the cell’s defenses against oxidative stress by stabilizing the cell membrane and preventing the propagation of ROS. However, the precise biochemical pathways and molecular interactions that underlie this protective mechanism are not yet fully understood (106).

In the retina, the activity of GCH1 is particularly important for protecting photoreceptor cells from light damage (107). Photoreceptor cells are particularly susceptible to oxidative damage due to their continuous exposure to light. the function of GCH1 is potentially important for maintaining retinal health and preventing certain ophthalmic diseases, such as age-related macular degeneration and certain types of retinal degenerative diseases.

Further research is needed to elucidate the detailed mechanisms by which GCH1 and its metabolites modulate lipid peroxidation and ferroptosis. Understanding these processes could provide valuable insights into the development of novel therapeutic strategies for diseases in which ferroptosis plays a significant role, such as neurodegenerative disorders, ischemic injury, and certain types of cancer.

## 3 Ferroptosis and other forms of cell death

Ferroptosis is a unique form of regulated cell death that relies on iron and sets itself apart from other forms such as apoptosis, programmed necrosis, and pyroptosis (108–110).

From a morphological perspective, ferroptosis is characterized by specific features, including the atrophy of mitochondria, a significant reduction and shriveling of mitochondrial cristae, an increase in membrane density, and instances of membrane rupture (111, 112). Notably, the nucleus often appears normal, and an increase in cell membrane density occurs. In contrast, apoptosis is characterized by a decrease in cell volume, chromatin condensation, nuclear fragmentation, the preservation of a complete membrane structure, and the formation of apoptotic bodies (113). Programmed necrosis is identified by an increase in cell size, simultaneous rupture of the plasma membrane, the generation of substantial cell debris, and, frequently, the formation of necrosome structures (114, 115). Pyroptosis involves the swelling of cells, the formation of pores on the plasma membrane, and the release of inflammatory mediators (116).

Biochemically and immunologically, ferroptosis is characterized by a significant reduction in the granular membrane potential, elevated levels of intracellular iron ions, a substantial increase in ROS levels, and intensified lipid peroxidation (117). It is also characterized by the release of damage-associated molecular patterns (DAMPs) and the promotion of an inflammatory response (118, 119). In apoptotic cells, DNA is cleaved into 180–200 base pair fragments, the mitochondrial membrane potential decreases significantly, phosphatidylserine flips from the inner to the outer membrane, and no endolysosomal release or inflammatory response occurs (119).

Programmed necrosis is characterized by the release of endolysates and DAMPs, which contribute to inflammation (120). In pyroptotic cells, inflammatory bodies such as the NLRP3 inflammasome can be observed (121). During pyroptosis, inflammatory caspases activate and cleave GSDMD, releasing its N-terminal fragment (GSDMD-N), which forms pores in bacterial membranes leading to their death without damaging the host cell (119).

These distinct characteristics of each form of cell death underscore the complexity of cellular demise and the importance of accurate identification for both diagnostic and therapeutic purposes. Understanding the biochemical and immunological signatures of ferroptosis, in particular, may provide novel avenues for intervention in diseases where this form of cell death plays a significant role.

### 3.1 Ferroptosis and apoptosis

Apoptosis, a form of PCD, is intricately governed by genetic mechanisms (122). It plays a vital role in maintaining tissue homeostasis, orchestrating immune and defense functions, and managing cellular damage associated with tumorigenesis (123).

A growing body of research indicates a close relationship between ferroptosis and apoptosis (124, 125). Under specific conditions, cells can transition from apoptosis to ferroptosis (126). This interplay can amplify the cellular susceptibility to apoptotic signals (127). Furthermore, the tumor suppressor protein P53, known for its role in halting the cell cycle and inducing apoptosis in tumor cells, can also trigger ferroptosis under certain circumstances (128). Both *in vivo* and *in vitro* investigations have shown that cells treated with metal organic network encapsulated with p53 plasmid (MON-p53) exhibit pronounced signs of ferroptosis and apoptosis. This dual action not only curbs tumor proliferation but also extends the lifespan of mice bearing tumors (129). Consequently, the convergence of ferroptosis and apoptosis pathways represents a promising avenue for therapeutic intervention in cancer treatment.

This emerging paradigm underscores the complexity of cell death mechanisms and highlights the potential for novel strategies that harness the interplay between ferroptosis and apoptosis (130). Targeting this combined pathway could represent a more comprehensive approach to managing tumor growth and potentially other diseases in which these cell death mechanisms are implicated. Future research is likely to explore the nuances of this interaction and develop targeted therapies that leverage the synergistic effects of ferroptosis and apoptosis.

### 3.2 Ferroptosis and programmed necrosis

Programmed necrosis is characterized by a cellular morphology resembling necrosis and involves a signaling pathway akin to apoptosis (131). Moreover, ferroptosis has been observed to coexist with programmed necrosis, particularly in the demise of neurons following hemorrhagic stroke (132). Studies have reported an increase in the mRNA levels of both ferroptosis and programmed necrosis markers during haem chloride-induced cell death (133).

In 2017, research conducted by Müller et al. (134) proposed that ferroptosis and programmed necrosis are complementary mechanisms of cell death. Within this framework, ACSL4, a key enzyme in lipid metabolism, was utilized as an indicator of ferroptosis, while mixed lineage kinase-like protein (MLKL), a protein involved in the execution of necrosis, served as a marker for programmed necrosis (135, 136). These findings indicated that a deficiency in ACSL4 resulted in the upregulation of MLKL, which sensitized cells to ferroptosis (137). This result suggests a compensatory relationship between the two pathways, where the suppression of one cell death mechanism can lead to the activation of the other.

Wulf Tonnus et al. (138) reported that NADPH, a critical coenzyme in cellular redox reactions, can diffuse freely between cells, potentially influencing both ferroptosis and programmed necrosis. The hypothesis posits that the consumption of NADPH during programmed necrosis may sensitize neighboring cells to ferroptosis (139). When cells undergo either programmed necrosis or ferroptosis, intracellular NADPH levels are reduced, potentially rendering adjacent cells more vulnerable to alternative cell death pathways (138). This perspective provides a more integrated view of how ferroptosis relates to and intersects with other PCD mechanisms, highlighting the complexity and interconnectivity of cell death processes.

### 3.3 Ferroptosis and autophagy

Autophagy, a process that relies on the lysosome for the degradation of cellular components, has been implicated in a spectrum of pathological conditions, including infectious diseases, immunological disorders, metabolic syndromes, and malignancies (140–142).

Recent investigations have revealed a novel role for autophagy in cancer cell death, showing that autophagy can initiate the breakdown of ferritin, consequently triggering ferroptosis (143). In fibroblasts and cancer cells, this form of iron-mediated autophagy has been shown to increase intracellular iron levels through the degradation of ferritin, further promoting ferroptosis (144).

Moreover, the deletion or inhibition of genes such as Atg5 and Atg7, which are essential for autophagy, has been reported to curtail ferroptosis induced by erastin (145, 146). This process is achieved by reducing both intracellular iron levels and the extent of lipid peroxidation (147). These discoveries shed light on the intricate relationships and cross-regulatory mechanisms between ferroptosis and autophagy, providing new insights into the complex network of cell death pathways and their interdependence (144).

## 4 Ferroptosis and ophthalmic disorders

**Figure 4:**
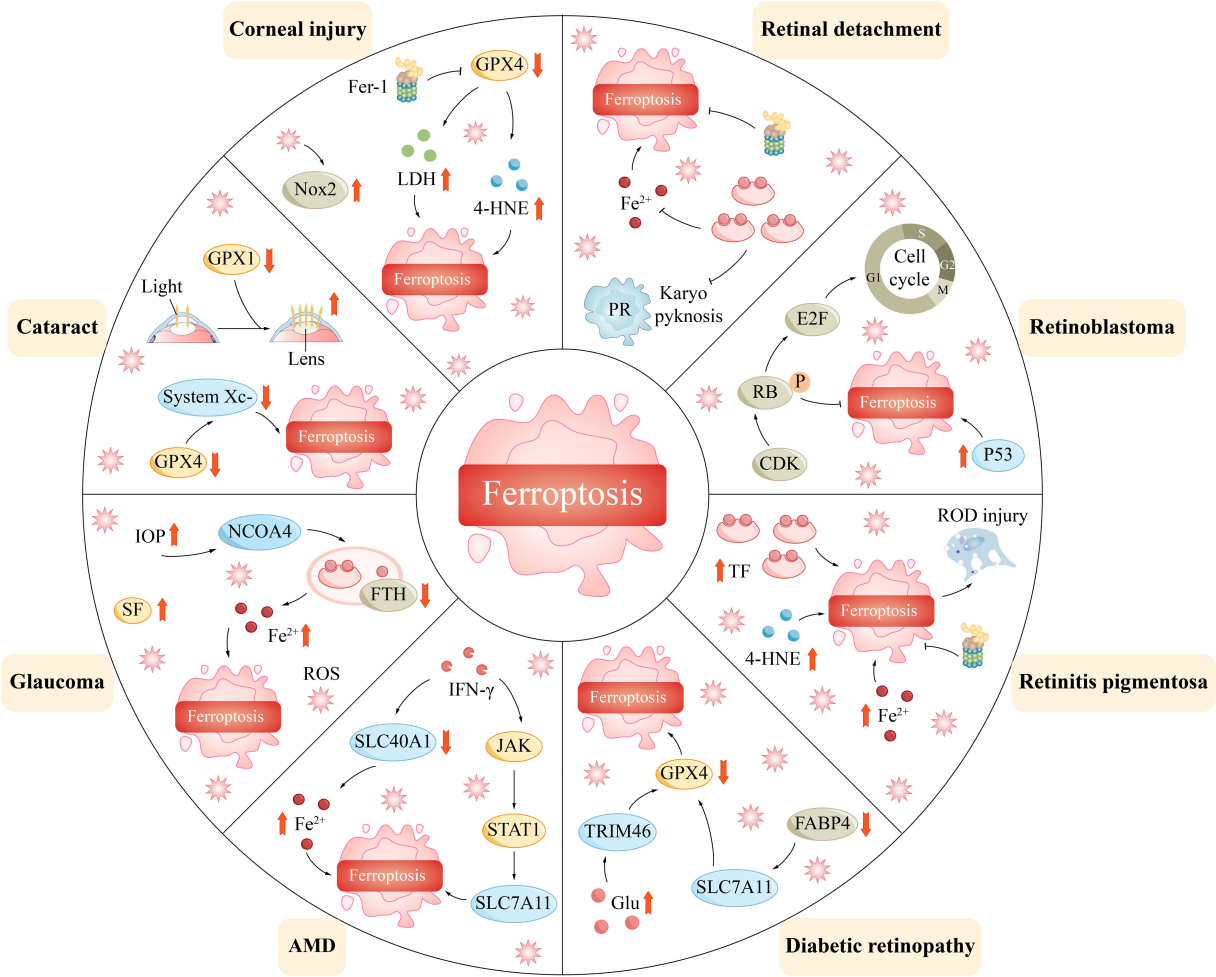
Ferroptosis mediates a variety of ophthalmic disorders.

Ferroptosis has been implicated in the progression of numerous diseases. Ferroptosis is particularly prevalent in ophthalmology and may be associated with corneal disease in the anterior segment, uveal disease in the middle segment, and retinal disease in the posterior segment of the eye (**Figure 4**). By exploring the regulatory mechanisms of ferroptosis, including the metabolism of iron ions, the production of reactive oxygen species, and the balance of antioxidant systems, we can obtain a better understanding of its role in ophthalmic diseases (**Table 1**).

**Table 1:** The mechanism of ferroptosis-related targets in ophthalmic diseases.

Ophthalmic diseases	Ferroptosis-related targets	Study subject	Mechanism	Reference
Corneal injury	GPX4	HCECs, C57BL/6 mice	Downregulation of GPX4 can promote the production of LDH and lipid peroxidation products such as 4-HNE	(148)
	Lipid ROS	Male ICR mice	High levels of ROS can upregulate the expression of NOX2, NOX4, VEGF, and MMPs	(149)
	Lipid ROS	HCECs	Accumulation of ROS leads to the upregulation of ferroptosis promoters and the downregulation of ferroptosis inhibitors	(150)
	GPX4	HCE-S cells	After exposure to Phenylarsine oxide, the expression of GPX4 in HCE-S cells is significantly reduced, indicating that Phenylarsine oxide induces ferroptosis mediated by lipid peroxidation in HCE-S cells	(151)
	GSH;GPX4	Male SD rats	siVEGF-TIL can inhibit oxidative stress, inflammation, and the expression of VEGF in vitro, and effectively maintain corneal transparency in vivo, accelerate epithelialization, and inhibit corneal neovascularization	(152)
	Ferrous ion	HCE-T cells	Exposure to CSE induces lipid peroxidation and the accumulation of ferrous ions within the lysosomal compartments	(153)
Cataracts	SIRT6	Male SD rats	Melatonin inhibits ferroptosis through the SIRT6/p-Nrf2/GPX4 and SIRT6/COA4/FTH1 pathways	(154)
	Nrf2, ferrous ion	Female C57BL/6J mice	Nrf2 may affect ferritin degradation by decreasing the expression of NCOA4	(155)
	GSK-3β/Nrf2	Female C57BL/6J mice	Targeting the balance of GSK-3β/Nrf2 can alleviate ferroptosis in LECs	(156)
	GPX4	LECs	Inhibition of IncRNA MEG3 by interacting with PTBP1 promotes the decay of GPX4 messenger RNA, accelerating cellular vitality and inhibiting ferroptosis	(157)
	GPX4	Male C57BL/6J mice, SD rats	Astaxanthin-mediated targeting of GPX4 may alleviate damage to human LECs by inhibiting ferroptosis and improving oxidative stress	(158)
	GPX4	ARCC patients	GPX4 downregulation enhanced LEC ferroptosis and decreased viability via RSL3 in SRA01/04 cells	(159)
	Hippo pathway	LECs	Downregulation of KRAs might restrain ferroptosis and affect Hippo pathway in cataract	(160)
	System Xc-, GPX4	LECs	System Xc inhibitor Erastin and GPX4 inhibitor RSL3 can induce ferroptosis in human LECs in vitro and in mouse corneal epithelium in vitro.	(161)
	Ferrous ion	LECs	The iron released from heme by HMOX1 may play a key role in increasing the sensitivity of human LECs to erastin	(162)
	GPX-1	C57BL/6 mice	Lipid peroxides are substrates for GPX-1, and lenses with elevated levels of GPX-1 activity can resist the cytotoxic effects of H2O2	(163)
Glaucoma	MAPK;SLC7A11;GPX4	Male SD rats	SB202190 can inhibit ferroptosis by regulating SLC7A11/GPX4 pathways, protecting retinal ganglion cells	(164)
	GPX4;FSP1;Nrf2	Male New Zealand rabbits	Overexpression of mitochondrial GPX4 rescued artesunate-induced lipid peroxidation and ferroptosis. FSP1 and Nrf2, which are also inhibited by artesunate.	(165)
	GSH, ROS	RGC-5 cells	GGT1 inhibits autophagy in RGC-5 cells by targeting GCLC, further suppressing the occurrence of cellular ferroptosis	(166)
	NCOA4-FTH1	Male C57BL/6 mice	The NCOA4-FTH1-mediated iron metabolism disorder and ferroptosis play a role in glaucomatous RGCs	(167)
	GPX4;System Xc-	Male Wistar rats, Male C57BL/6J mice	Treatment with ONC leads to a significant downregulation of GPX4 and system Xc- in rat retinas, accompanied by increased levels of peroxidized lipids and iron.	(19)
	Serum ferritin	Korean patients	Higher serum ferritin levels are associated with an increased likelihood of glaucoma	(168, 169)
AMD	Lipid ROS	ARPE-19;hRPE cells	Necrostatin-1 can inhibit the activation of RIPK1/RIPK3 and the accumulation of lipid ROS	(170)
	Ferrous ion	C57BL6 mice;ARPE-19 cells	Knocking down ZIP8 can significantly inhibit the ferroptosis induced by sodium iodate-induced oxidative stress in RPE cells	(171)
	Nrf2	661W cells;C57BL/6 mice	Melatonin inhibits the GSK-3β/Fyn-dependent nuclear translocation of Nrf2	(172)
	GPX4;FTH1	C57BL/6 mice	Overexpression of PEDF can upregulate the expression of GPX4 and FTH1	(173)
	Lipid ROS	ARPE-19;hTERT RPE-1 cells	Increasing the intracellular concentration of POS or DHA can enhance the susceptibility of the RPE to oxidative stress damage	(174)
	Ferrous ion, Hydroxyl radicals	ARPE-19	HIF exacerbates oxidative stress-induced ferroptosis in RPE cells	(175)
	Mitochondrial ROS	ARPE-19, C57BL/6J mice	CircSPECC1, mediated by m6A modification and sponging miR-145-5p, resists oxidative stress injuries and maintains lipid metabolism in RPE	(176)
	KEAP1, NRF2, Ferrous ion	C57BL/6J mice	Overexpression of HO-1 leads to a significant increase in Fe^2+^ levels in photoreceptor cells, promoting the excessive production of ROS	(177)
	Lipid peroxidation	ARPE-19, OMM-1	Ommochrome extract seem to be promising regarding protection against lipid peroxidation in healthy ocular cells	(178)
	Ferrous ion	C57BL/6J mice	Chac1 plays a key role in ferroptosis induced by oxidative stress by regulating GSH depletion.	(179)
	Nrf2	ARPE-19	GBE pre-treatment attenuates pro-oxidant stress and protects human RPE cells from oxidative injury by modulating ERK1/2-Nrf2 axis	(180)
	FTH1, GPX	C57BL/6J mice	Deregulated LCN2-iron axis triggers oxidative damage and lipid peroxidation in RPE cells	(181)
	Lipid ROS	C57BL/6J mice	CaPB effectively prevented the degeneration of RPE, significantly rescuing retinal structure and visual function	(182)
	Nrf2, SLC7A11, Ferrous ion	Male C57BL/6 mice, ARPE-19 cells	Nrf2-SLC7A11-HO-1 leads to RPE cell death and subsequent photoreceptor degeneration through the accumulation of ferrous iron ions and fatal oxidative stress	(183)
	GPX4, FSP1	HRPEpiC, ARPE-19 cells, Male C57BL/6J mice	Ferrostatin-1 significantly ameliorated the compromised GSH-GPX4 and FSP1-CoQ10-NADH signaling	(81)
	L-Ft	Male C57BL/6J mice	IVT FAC induced iron accumulation in Müller glia and myeloid cells, and the formation of lipidperoxidation products	(184)
	SLC40A1, System Xc-, Ferrous ion, GPX4	ARPE-19 cells	IFN-γ raises Fe^2+^ by inhibiting the iron exporter SLC40A1 and induces GSH depletion by blocking the System Xc- transporter.	(185)
	GSH	ARPE-19 cells	GSH depletion induces ferroptosis	(186)
	ferrous ion, ACSL4, System Xc-	C57BL/6J mice	Iron overload, GSH depletion, and mitochondrial damage cause the production of ROS, which, together with the activation of ACSL4, promote lipid peroxidation and thus induce ferroptosis.	(187)
	SLC7A11, Ferrous ion, Nrf2, GPX4	ARPE-19 cells, C57BL/6J mice	SLC7A11 overexpression induced resistance to erastin or RSL3-induced ferroptosis	(188)
	Ferrous ion, SLC7A11, GPX4	661W cells;Male SD rats	Light exposure significantly induced changes, including iron accumulation, mitochondrial shrinkage, GSH depletion, increased MDA, and decreased SLC7A11 and GPX4.	(189)
DR	GPX4, FTH1, ACSL4, TFRC	HRMECs	Under high glucose conditions, ferroptosis is associated with increased levels of ROS, lipid peroxidation, and iron content	(190)
	HMOX1	C57BL/6J mice	HMOX1 expression correlated with M2 macrophage infiltration and ferroptosis	(191)
	ROS	SD rats	Inhibition of AQP4 inhibits the ferroptosis and oxidative stress in Muller cells by downregulating TRPV4	(130)
	Ferrous ion, ROS	ARPE-19 cells	USP48 overexpression deubiquitinated SLC1A5 to elevate cell proliferation and suppress ferroptosis and oxidative stress	(192)
	ACSL4, TFR1, SLC7A11, GPX4, Ferrous ion	hRMECs	Overexpression of PIM1 inhibited the inflammatory response, oxidative stress, cell migration, and tube formation potential in hRMECs, whereas elevated tight junction protein levels.	(193)
	GPX4-YAP	DR patients, Male C57BL/6 mice, hRCECs	Pipecolic acid may impede the progression of DR by inhibiting the YAP-GPX4 signaling pathway	(194)
	GPX4, SLC7A11, ACSL4, FTH1, NCOA4	661W cells, Male C57BL/6J mice	The expression of GPX4 and SLC7A11 was downregulated, while the expression of ACSL4, FTH1, and NCOA4 was upregulated in the 661W cells cultured under HG conditions and in the photoreceptor cells in diabetic mice.	(195)
	SLC7A11, GPX4	hRMECs	Ferroptosis-related genes are significantly enriched in processes of ROS metabolism, iron ion reactions	(196)
	GPX4, FTH1, Ferrous ion	ARPE-19 cells, C57BL/6J mice	Sestrin2 inhibits ferroptosis by inhibiting STAT3 phosphorylation and ER stress	(197, 198)
	Nrf2, GPX4	ARPE-19 cells	Maresin-1 inhibits high glucose induced ferroptosis in ARPE-19 cells by activating the Nrf2/HO-1/GPX4 pathway	(199)
	GPX4, FSP-1	Male C57BL/6 mice, ARPE-19 cells	1,8-Cineole ameliorates DR by inhibiting RPE ferroptosis via PPAR-γ/TXNIP pathways	(200)
	Ferritin	ARPE-19 cells	BECN1, HERC2, ATG7, and BCAT2 might regulate ferritinophagy to influence its development and progression	(201)
	NRF2, GPX4, Ferrous ion	HRECs	Amygdalin treatment inhibited ferroptosis and oxidative stress in HG-stimulated HRECs by activating the NRF2/ARE signaling pathway	(202)
	GPX4	ARPE-19 cells	Ferrostatin-1 alleviates tissue and cell damage in DR by improving the antioxidant capacity of the Xc--GPX4 system	(203)
	FTH1	Tg (hb9: GFP) zebrafish	ACR could directly activate ferroptosis and impairs peripheral neurogenesis	(204)
	GPX4, SLC7A11, Ferrous ion	hRMVECs	25-hydroxyvitamin D3 inhibits oxidative stress and ferroptosis in retinal microvascular endothelial cells induced by high glucose through down-regulation of miR-93	(205)
	GPX4, GSH, Ferrous ion	DR patients	Compared with the normal group, GPX4 and GSH concentrations were significantly lower, and LPO, Fe^2+^, and ROS concentrations were significantly higher in DR patients	(206)
	ACSL4	Male SD rats	Glia maturation factor-β induces ferroptosis by impairing chaperone-mediated autophagic degradation of ACSL4 in early DR	(207)
	SLC7A11, GPX4	Male C57BL/6 mice, ARPE-19 cells	Downregulation of FABP4 alleviates lipid peroxidation and oxidative stress in DR by regulating peroxisome proliferator-activated receptor y-mediated ferroptosis	(208)
	Nrf2, GPX4	ARPE-19 cells	Astragaloside-IV alleviates high glucose-induced ferroptosis in RPE cells by disrupting the expression of miR-138-5p/Sirt1/ Nrf2	(209)
	GSH, Ferrous ion	ARPE-19 cells	Downregulation of Circular RNA PSEN1 ameliorates ferroptosis of the high glucose treated RPE cells via miR-200b-3p/cofilin-2 axis	(210)
	GPX4	HRCECs	TRIM46 aggravated high glucose-induced hyper permeability and inflammatory response in human retinal capillary endothelial cells by promoting IκBα ubiquitination	(211, 212)
	SLC1A5	ARPE-19 cells	miR-338-3p targeted the 3' untranslated regions (3'UTR) of SLC1A5 for its inhibition and degradation, and high glucose downregulated SLC1A5 by upregulating miR-338-3p in RPE cells.	(213)
RP	SLC7A11, GPX4, P53	661 W cells	Fructus Lycii and Salvia miltiorrhiza Bunge extract attenuate oxidative stress-induced photoreceptor ferroptosis	(50)
	ROS	ARPE-19 cells	TXNIP-Trx-TrxR redox pathway may participate in RPE dysfunction	(214)
	NRF2, GPX4, SLC7A11	rd10 mice, C57BL/6J mice	Qi-Shen-Tang alleviates retinitis pigmentosa by inhibiting ferroptotic features via the NRF2/GPX4 signaling pathway	(33)
	Ferritin, Ferrous ion	rd10 mice	RP are associated with altered iron homeostasis regardless of the primary insult	(215)
RB	GPX4, SLC7A11	A375, SK-Mel-28, and FO-1 cells	Hyperforin Enhances HO-1 Expression Triggering Lipid Peroxidation in BRAF-Mutated Melanoma Cells and Hampers the Expression of Pro-Metastatic Markers	(216)
	GPX4, FTH1, ACSL4	Y79 cells, Y79/DDP cells	USP14 might suppress the DDP resistance in RB by mediating ferroptosis	(217)
	Hippo pathway	Y79 cells, SO-RB50 cells, RB3823 cells	Expression of YAP suppresses cell proliferation and elevates the sensitivity of chemotherapy in RB cells through lipid-peroxidation induced ferroptosis	(218)
	GPX4, FTH1, SLC7A11, P62	ARPE-19, Y79 cells, Male BALB/c nude mice	Nuclear translocation of CTSB induces lysosomal stress, which eventually leads to ferroptosis	(219)
	FTH1, NCOA4	Y79 cells, WERI-Rb-1 cells	The anticancer potential of an itaconate derivative in RB cell relies on ferritinophagy-mediated ferroptosis	(220)
	P53	TKO cells	Deletion of RB genes enhanced ferroptosis	(221)
RD	Transferrin, Ferrous ion	RD patients, Male Long-Evans rats	TF decreases both apoptosis and necroptosis induced by RD	(222)

ACSL4, Acyl-CoA synthetase long-chain family member 4; AMD, Age-related macular degeneration; ARCCs, Age-related cortical cataracts; Chac1, GSH-specific γ-glutamylcyclotransferase 1; CSE, Cigarette smoke extract; DHA, Docosahexaenoic acid; DR, Diabetic retinopathy; FABP4, Fatty acid binding protein 4; FTH1, Ferritin heavy chain-1; GBE, Ginkgo biloba extracts; GCLC, Glutamate cysteine ligase catalytic subunit; GGT1, Gamma-glutamyl transpeptidase 1; GPX4, Glutathione peroxidase 4; HCECs, Human corneal epithelial cells; HCE-S, Human corneal epithelial cells; HIF, Hypoxia-inducible factor; HMOX1, Heme oxygenase 1; HRCECs, Human retinal capillary endothelial cells; IFN-γ, Interferon-gamma; KEAP1, Kelch-like ECH-associated protein 1; LECs, Lens epithelial cells; L-Ft, Ferritin light chain; MDA, Malondialdehyde; MMPs, Matrix metalloproteinases; Nrf2, Nuclear factor erythroid 2-related factor 2; ONC, Optic nerve crush; PEDF, Pigment epithelium-derived factor; POS, Photoreceptor outer segments; RB, Retinoblastoma; RD, Retinal detachment; RGC-5, Retinal ganglion cells; RGCs, Retinal ganglion cells; ROS, Reactive oxygen species; RP, Retinitis pigmentosa; RPE, Retinal pigment epithelium; SD, Sprague–Dawley; VEGF, Vascular endothelial growth factor.

### 4.1 Ferroptosis and corneal injury

Corneal injuries are medical conditions characterized by the disruption of the protective functions and structural integrity of the corneal epithelium due to various factors (223). This disruption can result in partial or complete loss of corneal epithelial cells. Clinical manifestations may include widespread punctate defects or erosions on the corneal surface, along with continuous detachment and damage to the epithelium (224). Additionally, varying levels of inflammation affecting the ocular surface may be present (225). In severe instances, such injuries can lead to pathological alterations in the corneal stroma, potentially impacting visual acuity (226).

Ferroptosis research, triggered by corneal injury, is attracting medical research interest. Particularly in cases of corneal trauma, damage to the corneal epithelial layer is notably prevalent. When the cornea sustains an injury, it initiates a series of intricate biochemical reactions, with oxidative stress being a particularly prominent factor (151, 227).

GPX4, an essential player in ferroptosis, is integral to maintaining the oxidative balance, ensuring cell survival, and facilitating the wound healing process in corneal epithelial cells (228). Research conducted by Sakai et al. (148) reported that a reduction in GPX4 levels, achieved through the transfection of GPX4 siRNA into the human corneal epithelial cell line HCEC, could lead to an increase in lipid peroxidation products such as lactate dehydrogenase (LDH) and 4-hydroxynonenal (4-HNE), thus triggering ferroptosis. Furthermore, the application of Fer-1, an inhibitor of ferroptosis, was found to mitigate cellular damage associated with the knockout of the GPX4 gene (229). Additional studies have indicated that mice with partial knockout of GPX4 exhibit slower corneal wound healing after epithelial injury than their wild-type counterparts, underscoring the significance of GPX4 in the repair process following corneal injury.

Corneal alkali burns represent an exceedingly severe form of corneal chemical injury that inflicts substantial damage on the ocular surface, with a particular emphasis on the cornea. Addressing this type of injury is complex, and the prognosis is often unfavorable, frequently resulting in a significant visual impairment or even blindness. During corneal alkali burn injury, ROS production is substantially increased, which can increase the expression of genes such as Nox2, Nox4, VEGF, and MMP. This upregulation contributes to further corneal damage and encourages the development of new blood vessels (149). Wang et al. (150) reported that ferroptosis, induced by lipid peroxidation, plays a pivotal role in the injury caused by alkali burns, along with ROS. The pathological changes linked to ferroptosis can be partially reversed by treatment with Fer-1. However, due to the hydrophobic nature of Fer-1, its direct application in clinical settings is not feasible. Consequently, Wang et al. (150) have engineered a liposomal delivery system for Fer-1 (Fer-1-NPs) to enhance its bioavailability and augment its therapeutic effects on corneal cloudiness and neovascularization.

In addition to the presence of ferroptosis during the process of corneal alkali burn, excessive ROS and reactive nitrogen species (RNS) may also be produced in corneal inflammation or corneal injury caused by trauma (230). These substances can promote the release of iron ions and lipid peroxidation, thereby triggering ferroptosis (231). Furthermore, corneal surgery or infection may also induce similar oxidative stress reactions, which can subsequently impact the metabolism of iron ions and the cellular redox state.

The collective findings from these studies highlight the critical role of ferroptosis in corneal injury and suggest that its inhibition could be beneficial for corneal preservation (152, 232). The development of pharmaceuticals based on the understanding of ferroptosis offers promising avenues for treating corneal injuries (233). Moreover, these investigations provide a substantial theoretical framework and empirical data that can inform the creation of novel therapeutic approaches for treating corneal injuries. Overall, an in-depth understanding of the ferroptosis mechanism is anticipated to pave the way for more efficacious treatment strategies for corneal injuries, providing renewed hope to those suffering from keratopathy.

In summary, the study of ferroptosis in the context of corneal injury represents a multifaceted and significant area of research. A thorough investigation of the roles played by key elements, such as GPX4, ROS, and lipid peroxidation, in the ferroptosis process, coupled with the development of potent ferroptosis inhibitors, can yield innovative strategies for treating corneal injuries. These research outcomes are also likely to serve as valuable references for addressing other related medical conditions. As scientific and technological advancements continue to progress, further breakthroughs and advancements in the field of corneal injury treatment will occur.

### 4.2 Ferroptosis and cataract

Cataract, a prevalent eye disorder, is identified by clouding of the ocular lens, which impairs visual acuity (234). This ailment can impact either a single eye or both simultaneously. Affected individuals often report a range of symptoms, including but not limited to, hazy vision, heightened nearsightedness, the occurrence of double vision, the appearance of light halos, photophobia, and diminished visual capacity in low-light conditions (235). Such symptoms can significantly impact daily activities, such as driving and reading, and can also hinder the ability to discern faces. Patients with critically impaired vision have an elevated risk of falls and an increased propensity for depressive symptoms (236). Cataract accounts for half of the global blindness cases and is responsible for 33% of visual impairments worldwide (237).

Recent scientific investigations have revealed that the ageing process can disrupt the redox equilibrium of the lens, leading to a decrease in the activity of enzymes linked to the glutathione synthesis pathway (238). This change results in a reduction in GPX activity, an increase in the accumulation of ROS, and an increase in lipid peroxidation byproducts (239). These discoveries point towards the potential involvement of ferroptosis in the genesis of cataracts.

In a study by Reddy and colleagues (163), a noted increase in the scattering of light within the nuclei of lenses from mice that were genetically deficient in GPX1 was documented. Over time, these mice exhibited signs of age-related cataracts, a phenomenon not observed in the wild-type control group. Wei et al. (161) reported that lens epithelial cells are susceptible to ferroptosis when exposed to inducers of this process. The level of GSH was identified as a pivotal determinant of cell vulnerability. Additionally, Wei et al. (161) observed that the sensitivity of lens epithelial cells to ferroptosis inducers increases with the extent of cellular aging.

Hydroxyl radicals are highly reactive and can attack PUFAs in the cell membrane, initiating lipid peroxidation (240). This process leads to damage and dysfunction of the cell membrane structure, impacting the normal physiological activities of lens cells. Apart from lipids, hydroxyl radicals can also target proteins and nucleic acids, resulting in protein cross-linking and DNA damage (241). These damages can impair the cell’s repair mechanisms and the stability of genetic information, ultimately influencing cell survival. Damage to lens cells can cause the accumulation and precipitation of proteins within the cells, diminishing the transparency of the lens and contributing to the development of cataracts.

Further analysis of the transcriptome revealed that in older cells, GPX4 expression was downregulated, System Xc- function was suppressed, and genes involved in iron ion uptake were upregulated, while genes responsible for iron ion export were downregulated. These findings indicate that ferroptosis could be instrumental in the progression of cataracts (154). Elucidating the relationship between ferroptosis and cataract development may pave the way for innovative preventative strategies and therapeutic approaches for cataract treatment.

### 4.3 Ferroptosis and glaucoma

Glaucoma encompasses a collection of eye disorders linked to abnormal levels of intraocular pressure (242). This condition has the potential to inflict damage on the optic nerve, leading to a reduction in the visual field and a loss of vision (243). The clinical manifestations of glaucoma can include a range of symptoms such as a decrease in visual acuity, discomfort or pain in the eyes, moderate enlargement of the pupils, redness and swelling of the ocular area, and nausea (244). Typically, glaucoma first affects peripheral vision; without timely intervention, it may progress to the central visual field, potentially resulting in irreversible blindness (245).

Recent scientific findings suggest that iron ions could be instrumental in injury to retinal ganglion cells (RGCs). Lin et al. (168) documented a positive correlation between increased levels of serum ferritin and the incidence of glaucoma. This correlation implies that disruptions in iron metabolism might be intertwined with the pathogenesis of the disease. Building on this information, Yao et al. (167) showed that pathological increases in intraocular pressure (IOP) can disrupt iron homeostasis, leading to an excessive accumulation of iron ions (Fe^2+^) in the retina, particularly affecting the RGC layer. This overabundance of iron ions compromises the retinal redox balance, triggering ferroptosis in RGCs. The administration of deferiprone, an iron ion chelator, has been found to significantly reduce iron ion levels in the retina, effectively inhibiting RGC death.

Moreover, comprehensive studies have indicated that pathologically elevated IOP might also expedite the degradation of ferritin by enhancing NCOA4-mediated ferritin autophagy (246). This process leads to the release of a substantial amount of previously bound iron ions into the cell. Once ferritin is degraded, the iron ions it had sequestered are released, intensifying the oxidative stress response and causing harm to RGCs. Knockdown of the NCOA4 gene can effectively prevent ferritin degradation and curtail the release of iron ions, thereby significantly diminishing iron ion levels and mitigating RGC damage (247).

The aforementioned research suggests that ferroptosis can contribute to the demise of RGCs through diverse pathways, thereby influencing the advancement of glaucoma. Recent studies have revealed that the reduction of GPX4 expression triggers ferroptosis in nerve cells, indicating that ferroptosis may further aggravate the progression of glaucoma by targeting optic nerve damage (248).

These insights not only illuminate the potential role of iron ions in the development of glaucoma but also present a novel perspective for therapeutic approaches (249). Regulating iron homeostasis, especially by reducing iron ion accumulation due to pathological IOP, could represent a groundbreaking strategy for glaucoma management (250). Future research may explore the regulatory mechanisms of iron metabolism and investigate how these pathways can be targeted by pharmaceuticals or other treatments. These investigations could lead to the development of more effective treatment plans for glaucoma patients. Collectively, these studies may pave the way for innovative preventative measures and therapeutic interventions for glaucoma with the potential to slow or even prevent the progression of this debilitating eye disease.

### 4.4 Ferroptosis and age-related macular degeneration

Age-related macular degeneration (AMD) is a complex ocular disorder that occurs with ageing and is characterized by a deterioration in the macula structure and function (251). The macula, which is situated at the retina’s core, is pivotal for sharp central vision (252).

Recent in-depth examinations of retinal pigment epithelial (RPE) cells revealed that interferon-γ (IFN-γ) can trigger ferroptosis in these cells, contributing to the progression of AMD (185). The process is intricate and includes several stages: it starts with the suppression of the iron transporter SLC40A1, which results in an increase in intracellular iron (Fe^2+^) levels; this step is followed by the obstruction of System Xc−, which leads to a depletion of GSH within the cell. This process also involves the upregulation of acyl-CoA synthetase long-chain family member A (ACSLA) and 5-lipoxygenase (5-LOX), which promote lipid peroxidation (253). Finally, the downregulation of SLC7A11, GPX4, and GSH occurs through the activation of the JAK1–2/STAT1/SLC7A11 signaling pathways, stimulating ferroptosis (185).

In addition to IFN-γ, the pigment N-retinol-N-retinoethanolamine (A2E), a distinctive fluorophore within RPE cells, has also been implicated in ferroptosis (254). Through comprehensive transcriptome profiling, Scimone and colleagues noted the upregulation of A2E expression and the concomitant downregulation of SLC7A11 in H-RPE cells upon blue light exposure. This change curtailed the biosynthesis of GSH, further propagating ferroptosis in RPE cells.

Ferroptosis impacts not only RPE cells but also traditional AMD models (255). Liu and associates (256) analyzed sodium iodate (SI)-induced AMD cellular models and discovered that SI, while not influencing GPX4 activity, could deplete intracellular GSH, liberate Fe^2+^ from liposomes, increase intracellular ROS production, and increase lipid aggregation, consequently precipitating ferroptosis in ARPE-19 cells. Lee et al. (257) also detected that SI could provoke the generation of mitochondrial ROS, promoting ferroptosis in RPE cells.

Tang et al. (183) reported that ferroptosis induced by SI in RPE cells is linked to the Nrf2/SLC7A11/HO-1 pathway, with heightened HO-1 expression precipitating the upregulation of the TFR and the downregulation of SLC40A1, culminating in iron ion aggregation. The liposomal accumulation of Fe^2+^ can then increase HO-1 expression, perpetuating ferroptosis. The suppression of HO-1 or the application of the HO-1 inhibitor ZnPP has been shown to ameliorate the detrimental effects of ferroptosis on the morphology and functionality of RPE cells and photoreceptors (258).

Ferroptosis in photoreceptors also contributes significantly to the aetiology of AMD, particularly the dry form (50). Within the visual cycle, the regeneration of 11-cis-retinoic acid retinol (atRAL) between photoreceptors and the RPE is crucial for sustaining visual acuity (259). ATP-binding cassette transporter A4 (ABCA4) facilitates the transport of atRAL from the outer segment discs of photoreceptors to the cytoplasm, while all-trans retinol dehydrogenase 8 (RDH8) catalyses the conversion of atRAL back to all-trans retinol (260). In mouse models deficient in the Abca4 and Rdh8 genes, a deficiency in the clearance of photoreceptor outer segment discs, thinning of the retinal photoreceptor layer postlight exposure, the accumulation of atRAL in the retina, diminished GSH levels, aberrant expression of the lipid metabolism-related proteins COX2 and ACSLA, and heightened lipid peroxidation—indicators of ferroptosis—are observed (187). Subsequent studies have validated that atRAL can incite ferroptosis in the cone cell line 661W by impeding System Xc−, increasing Fe^2+^ levels, and amplifying mitochondrial ROS production.

Tang et al. (189) confirmed these findings, showing that light exposure can precipitate the degeneration of retinal photoreceptor cells through ferroptosis in 661W cells and male Sprague−Dawley rats. Illumination significantly diminishes the viability of photoreceptor cells and induces pro-ferroptosis alterations, such as iron buildup, mitochondrial condensation, GSH depletion, increased malondialdehyde (MDA) levels, and reduced expression of the SLC7A11 and GPX4 proteins.

The decrease in glutathione and GPX4 may result in the retinal cells’ ineffectiveness at efficiently eliminating lipid peroxides, thereby impairing the visual function of these cells and worsening AMD (81). The abnormal build-up of ROS and other oxidative stress molecules inflicts damage on the retinal cells, influencing the transmission and processing of visual signals (261). Moreover, as AMD is a neurodegenerative disease, ferroptosis could potentially hasten the progression of AMD by advocating the demise of nerve cells.

Ferristatin-1 has been shown to attenuate the impact of light on ferroptosis, ameliorate photoreceptor atrophy and ferroptosis, curb neuroinflammation, and protect the retinal structure and function from the effects of light (51, 189). Collectively, these insights provide innovative avenues for leveraging the mechanisms of ferroptosis in the prevention and treatment of retinal degeneration diseases, including AMD.

### 4.5 Ferroptosis and diabetic retinopathy

Diabetes mellitus is widely acknowledged as the primary cause of diabetic retinopathy (DR) (262). Persistently high blood sugar levels inflict damage on retinal vascular endothelial cells, resulting in a spectrum of retinal lesions, such as microaneurysms, hard exudates, cotton-wool spots, neovascularization, vitreous proliferation, macular oedema, and, in extreme cases, retinal detachment (263).

Recent research has revealed the crucial role of ferroptosis in the progression of DR, where it contributes to the disease by impairing the integrity of capillary endothelial cells. Uric acid, a significant biomarker for type 2 diabetes, influences serum uric acid levels through the TRIM46 gene, which exhibits increased expression in diabetic individuals (264). Under high-glucose conditions, the expression of TRIM46 in human retinal capillary endothelial cells (HRCECs) increases and correlates with the extent of cell death (212). Studies have indicated that TRIM46 overexpression facilitates the ubiquitination and subsequent degradation of GPX4 in HRCECs, thereby promoting ferroptosis.

Ferroptosis affects not only the microvasculature but also RPE cells. Research by Liu and colleagues has shown that in diabetic rat models, hyperglycemia induces the secretion of GMFB by Müller cells, and elevated levels of GMFB can impair retinal function by altering the quantity and connectivity of RPE cells (207). GMFB is capable of hindering lysosomal assembly and increasing alkalinity, which diminishes lysosomal functionality. This reduction in function impedes the autophagic degradation of ACSLA, leading to its accumulation and exacerbating lipid peroxidation in ARPE19 cells. This process results in a decrease in mitochondrial activity and the eventual induction of ferroptosis.

In addition to GMFB, fatty acid binding protein 4 (FABP4) has been shown to alleviate lipid peroxidation and oxidative stress associated with DR by modulating PPARγ-mediated ferroptosis (208). FABP4 is instrumental in sustaining the glucose and lipid balance and serves as an independent prognostic marker for patients with type 2 diabetic retinopathy.

PPARγ, a crucial modulator of fatty acid storage and glucose metabolism, regulates the expression of the FABP4 gene Conversely, FABP4 can initiate the ubiquitination and subsequent proteasomal degradation of PPARγ. In a streptomycin-induced diabetic mouse model, a marked increase in the expression of both the FABP4 and PPARγ proteins was observed in retinal tissue. Suppressing FABP4 has been shown to reverse the downregulation of the SLC7A11 and GPX4 proteins, thereby mitigating ferroptosis in RPE cells (208).

Furthermore, Tang and colleagues confirmed that RPE cells subjected to high-glucose conditions undergo ferroptosis (209). Persistent hyperglycemia intensifies oxidative stress, precipitating structural and functional alterations in retinal neurons (265). This degeneration characterizes the early neural damage in DR, potentially resulting in vision loss and other visual impairments. Oxidative stress induced by iron overload may also contribute to the manifestation of macrovascular complications of diabetes, including atherosclerosis and cardiovascular diseases (266). Within the context of DR, these changes could cause damage to and dysfunction of the retinal microvasculature. Therefore, inhibiting ferroptosis to curtail oxidative damage in the retina is a novel therapeutic approach for the management of diabetic retinopathy.

In summary, the pathogenesis of diabetic retinopathy involves a complex interplay of factors, including ferroptosis, uric acid metabolism, GMFB, and FABP4. These findings provide new insights into the intricate mechanisms underlying DR and present potential targets for future therapeutic interventions.

### 4.6 Ferroptosis and retinitis pigmentosa

Retinitis pigmentosa (RP), an inherited disorder, results in progressive degeneration of the retina, leading to night blindness, retinal atrophy, pigment deposition, and constriction of the visual field, potentially culminating in total blindness (267). The advancement of RP significantly diminishes the quality of life for those affected, underscoring the importance of investigating its underlying pathological processes to identify effective therapeutic approaches (268).

In the study of RP, researchers have focused on the expression levels of genes involved in iron metabolism, as well as the levels of iron and oxidative damage within the retina. In the rd10 mouse model, which exhibits rapid progression of retinal degeneration, a notable increase in the expression of several key proteins related to iron transport and storage, such as transferrin, ceruloplasmin, ferritin, and the transferrin receptor, was observed (215). Concurrently, an increase in the total iron content and the amount of iron bound to ferritin within the retina was observed. Moreover, the level of 4-hydroxy-2-nonenal (4-HNE), an indicator of lipid peroxidation, was significantly elevated, suggesting a strong correlation between excess iron and retinal damage (215).

Further research has established a connection between ferroptosis—the form of regulated cell death associated with iron metabolism and oxidative stress—and cell death induced by sodium iodate in ARPE-19 cells, highlighting the relevance of ferroptosis to the progression of RP (269). This discovery provides a novel perspective on the pathogenesis and potential treatment of this disease.

Mitochondrial dysfunction can result in the leakage of iron ions from the mitochondria into the cytoplasm. These iron ions react with ROS to generate hydroxyl radicals via the Fenton reaction (270). The accumulation of iron ions coupled with the production of hydroxyl radicals escalates the level of oxidative stress (271). Notably, oxidative stress is a pivotal factor in the onset of RP, which can inflict damage on photoreceptor cells, leading to cellular dysfunction and death (272). Moreover, the oxidative stress and cellular damage brought about during the process of ferroptosis can trigger inflammatory responses. The subsequent release of inflammatory mediators further impairs retinal cells, exacerbating the pathologic progression of RP (273).

Subsequent studies using RP models have revealed the efficacy of various iron homeostasis regulators in mitigating ferroptosis in photoreceptor cells and in protecting against photoreceptor degeneration. Notably, agents, such as zinc deferoxamine (274), the iron-chelating drugs (275) VK28 and VAR10303, the iron chelator deferiprone (276), and the ferroptosis inhibitors deferoxamine and hepstatin-1 (183), have all been proven to be effective in slowing ferroptosis. These findings offer a fresh scientific rationale for targeting iron homeostasis in therapeutic strategies for RP and propose innovative avenues for its management.

In conclusion, maintaining iron homeostasis is crucial in the pathogenesis of RP, with alterations in iron metabolism-related genes and iron levels being intricately linked to retinal damage. The utilization of iron chelators and inhibitors of ferroptosis represents innovative therapeutic avenues for RP and is anticipated to emerge as a promising treatment strategy in the future. These insights not only enhance our understanding of the pathological mechanisms of RP but also pave the way for the development of novel therapeutics, providing hope for more efficacious treatment solutions for individuals with RP.

### 4.7 Ferroptosis and retinoblastoma

Retinoblastoma (Rb), a malignant neoplasm originating from the precursor cells of the retinal photoreceptors, predominantly affects children under the age of three (277). This tumor presents with a variety of clinical signs and symptoms, which may affect one or both eyes and include conjunctival hyperemia, ocular oedema, corneal swelling, vitreous body turbidity, increased intraocular pressure, and ocular misalignment (278).

Delving into the pathogenesis of Rb, genetic alterations, particularly mutations in the p53 gene and the loss of the RB1 gene, have been pinpointed as pivotal for disease development. The p53 gene, known for its tumor-suppressive properties, modulates the phosphorylation of the RB protein by influencing cyclin-dependent kinases (CDKs), thereby regulating the E2F family of transcription factors, which are integral to the progression of the cell cycle. Disruptions in this regulatory system due to p53 mutations or RB1 gene deletions can lead to the abnormal activation of genes that drive the cell cycle and heightened vulnerability to ferroptosis (221).

Ferroptosis, an emerging form of regulated cell death, is closely linked to disruptions in iron metabolism and increased oxidative stress (279, 280). A significant challenge in Rb treatment is the potential for tumor cells to develop resistance to chemotherapy drugs. However, recent research has indicated that inducing autophagy-dependent ferroptosis can effectively counteract drug resistance in retinoblastoma cells. Itaconic acid derivatives have been shown to enhance ferroptosis by stimulating autophagy, thereby diminishing the activity of caspases, key mediators of apoptosis, and reversing the efficacy of chemotherapeutic agents such as carboplatin, etoposide, and vincristine (220).

By inducing selective ferritinophagy, a process that specifically degrades ferritin within the cell, the reserves of intracellular iron ions can be diminished, thereby heightening the cell’s sensitivity to ferroptosis. 4-Octyl itaconate (4-OI) is a metabolite capable of inducing ferritinophagy-dependent ferroptosis (281). Future studies could explore the possibility of 4-OI inducing ferritinophagy-dependent ferroptosis in Rb.

Furthermore, the therapeutic potential of ferroptosis has been noted in the treatment of other malignancies. In the context of advanced hepatocellular carcinoma (HCC), the RB protein has been shown to facilitate ferroptosis when exposed to sorafenib, thereby hastening the oxidative necrosis of cancer cells (282).

By synthesizing these findings, modulating ferroptosis is evidently a novel therapeutic strategy in oncology. By meticulously controlling the onset of ferroptosis, tumor cell resistance to chemotherapeutic agents can be significantly diminished and the death of cancer cells can be expedited. This approach is particularly pertinent to Rb, given that it is commonly diagnosed in early childhood, necessitating treatments with heightened sensitivity and responsiveness.

Consequently, the modulation of ferroptosis introduces a novel therapeutic perspective for Rb and may also offer novel insights into the treatment of other types of cancer. A deeper understanding of the molecular underpinnings of ferroptosis, coupled with the development of targeted modulators, holds promise for the delivery of more efficacious and targeted treatment regimens for patients afflicted with Rb. This area represents an auspicious frontier with the potential to bring about transformative advances in cancer therapy.

### 4.8 Ferroptosis and retinal detachment

Retinal detachment (RD) is a significant ophthalmological condition characterized by the separation of the retina from the underlying RPE and choroid (283). The formation of retinal tears or holes is a prevalent cause of this detachment, which, if neglected, can result in permanent vision impairment (284).

Contemporary medical research has detected an increase in iron concentrations and total iron-binding capacity within the vitreous and subretinal fluid as the duration of retinal detachment in patients increases (285). An examination of retinal tissue sections from individuals with RD revealed substantial iron accumulation within the RPE. This accumulation implies a potential correlation between aberrant iron ion levels and the pathogenesis of RD.

According to more in-depth experimental investigations, the administration of transferrin can efficiently mitigate the accumulation of iron ions in the RPE of rodent models of RD (285). This intervention also prevents the nuclear condensation of photoreceptor (PR) cells, thereby providing substantial protection against PR cell damage and reducing the incidence of ferroptosis (222). These findings indicate that iron ions may be partially responsible for PR cell damage during the onset of RD.

The discovery of the role of iron in RD opens the possibility of employing iron chelators as a novel therapeutic approach to diminish the damage associated with RD. The strategic use of iron chelators post-RD could reduce PR cell damage and enhance postoperative visual rehabilitation in patients. This approach introduces a novel avenue of research for the management of the RD prognosis.

The RPE cell layer provides support and nourishment for photoreceptor cells, and the integrity of its tight junctions is crucial for the structure and function of the retina (286). Ferroptosis could disrupt these tight junctions, leading to fluid permeation between the retinal layers, which could compromise the stability of the retina (287). RPE cells are responsible for phagocytosing and digesting the membrane discs of the outer segments of photoreceptor cells, a critical component of the visual cycle. Ferroptosis could possibly affect the phagocytic capability of RPE cells, disrupting the normal renewal of photoreceptor cells. This could potentially hinder the reattachment process and impede visual recovery (170).

In conclusion, iron ions are pivotal contributors to the pathophysiology of RD. The use of iron chelators is a promising therapeutic strategy for RD management. Subsequent studies may further elucidate the interplay between iron metabolism and the development of RD, as well as the prospective therapeutic utility of iron chelators in treatment protocols. This line of research could not only enhance the therapeutic outcomes for RD patients but also offer innovative treatment paradigms for a spectrum of ophthalmic conditions.

## 5 Therapeutic approaches targeting ferroptosis in ophthalmic disorders

Recently, significant progress has been achieved in the study of ferroptosis, revealing a diverse array of drugs associated with this process. These drugs include compounds derived from natural sources to synthetic small-molecule inhibitors and inducers. These discoveries provide fresh insights for mechanistic studies and potential therapeutic strategies for ferroptosis treatment.

In corneal injury, reducing the levels of GPX4, an antioxidant enzyme in corneal epithelial cells, leads to increased lipid peroxidation and ferroptosis. Fer-1, which inhibits ferroptosis, has been shown to decrease cell damage due to reduced GPX4 levels, suggesting its utility in corneal recovery. Fer-1 has been shown to partially ameliorate the associated pathology, especially in severe corneal injuries such as alkali burns, where ferroptosis significantly impacts tissue damage, as reported by Wang et al. (150). Given the hydrophobic properties of Fer-1, which can hinder its clinical use, researchers have developed a liposomal formulation (Fer-1-NPs) to improve its therapeutic efficacy. This development implies that drugs targeting ferroptosis could offer novel treatment options for corneal injuries, potentially enhancing treatment outcomes for keratopathy and similar conditions.

Cataract treatment can benefit significantly from understanding the association with ferroptosis, which affects the lens’s redox balance and the activity of enzymes in the glutathione pathway (239). Consequently, these changes lead to decreased GPX activity along with the accumulation of ROS and lipid peroxidation byproducts. In a study, mice deficient in GPX1 exhibited signs of age-related cataracts attributed to heightened light scattering within the lens nucleus (163). Additionally, lens epithelial cells are susceptible to ferroptosis, and the level of GSH plays a crucial role in determining their vulnerability (161). Gaining insights into this connection could pave the way for innovative preventative strategies and therapeutic interventions for cataract management.

In glaucoma, the physiological function of RGCs is influenced by iron ions and ferritin. Elevated serum ferritin levels are associated with the risk of glaucoma, and increased IOP can lead to the excessive accumulation of iron in the retina, triggering ferroptosis in RGCs (167, 168). Deferiprone, an iron chelator, has been reported to reduce iron levels and protect RGCs. Moreover, high IOP may induce ferritin degradation, resulting in the release of iron ions and exacerbating RGC injury. However, the inhibition of ferritin degradation by knocking down the NCOA4 gene attenuates RGC damage caused by high IOP (167). These findings indicate that targeting ferroptosis could serve as a novel therapeutic strategy for glaucoma and may offer innovative options for slowing disease progression.

Tang and colleagues developed an innovative iron chelator known as CaPB to address the issue of elevated iron levels in RPE cells and Bruch’s membrane in patients with AMD (182). This compound is characterized by its high biocompatibility and minimal cytotoxic effects, enabling it to selectively target and reduce intracellular bivalent iron concentrations. Additionally, CaPB mitigates oxidative stress by suppressing the expression of specific genes, thus potently inhibiting ferroptosis. This discovery suggests that the modulation of ferroptosis could emerge as a novel therapeutic approach for the treatment and prevention of AMD, providing a promising avenue for the development of new pharmaceutical interventions.

Ferroptosis plays a significant role in the progression of DR by impairing retinal endothelial cells and is associated with the upregulation of the TRIM46 gene in diabetic patients (212, 264). Inhibiting the effect of this gene on GPX4 degradation could alleviate this condition. Furthermore, interventions targeting FABP4 in PPARγ-mediated ferroptosis may decrease the oxidative stress and lipid peroxidation linked to DR (208, 209). These findings indicate that therapies concentrating on ferroptosis pathways could represent novel treatment options for diabetic retinopathy, underscoring the potential of molecular interventions in combating diabetic complications.

RP studies have highlighted the significance of iron metabolism-related genes and oxidative damage in the retina. Therapies targeting iron homeostasis, including zinc deferoxamine, VK28, VAR10303, deferiprone, and ferroptosis inhibitors such as deferoxamine or hepstatin-1 (183, 274–276), have shown promise in slowing photoreceptor cell degeneration in individuals with RP. These findings underscore the importance of iron management in treating RP and suggest innovative approaches to address this degenerative eye disease, potentially leading to more effective treatments for affected individuals.

Targeting ferroptosis in Rb represents a pioneering therapeutic approach. Central to Rb are genetic mutations in the p53 and RB1 genes, which heighten cell susceptibility to ferroptosis (221). Inducing ferroptosis through autophagy has the potential to overcome drug resistance in Rb cells, and itaconic acid derivatives can enhance chemosensitivity to agents such as carboplatin and etoposide (220). The role of the RB protein in promoting ferroptosis has also been observed in HCC, indicating the broader applicability of this strategy.

Elevated iron levels in the vitreous and subretinal fluid of RD patients suggest a link to RD pathogenesis. In rodent models, transferrin administration reduced iron accumulation in RPE cells, prevented photoreceptor cell damage, and decreased ferroptosis, indicating the role of iron in cell damage during RD (222). The potential use of iron chelators post-RD could protect photoreceptor cells and enhance visual rehabilitation, suggesting a novel therapeutic strategy.

Targeting ferroptosis for the treatment of ophthalmic diseases has emerged as a significant area of research. However, current targeted therapies face several challenges. For instance, the cornea’s structure complicates drug delivery, and traditional eye drops have low bioavailability due to factors such as tear dilution and blinking. Nanomedicine drug delivery systems offer an effective strategy for addressing the challenges of ocular drug delivery. By enhancing corneal penetration, achieving sustained drug release, and improving bioavailability, nanomedicine could potentially enhance the therapeutic effect of treatments for eye diseases, thereby improving patients’ quality of life.

The eye’s immune system is distinct from that of other body parts and possesses a unique immune privilege. This means that the eye can avoid triggering a robust immune response, which is crucial for protecting the eye from damage. However, it also implies that therapeutic drugs need to be specially designed (combining targeted drugs with immunosuppressants) to prevent unnecessary inflammation or immune reactions.

Currently, there is limited clinical research on targeting ferroptosis for the treatment of ophthalmic diseases, with most studies focusing on animal experiments. Given the differences between disease models and human biology, understanding the absorption, distribution, metabolism, and excretion of drugs in the human eye is vital for effective treatment.

Finally, as scientists deepen their research on the mechanism of ferroptosis, we anticipate a more comprehensive understanding of ferroptosis as research progresses. This will likely result in more therapeutic targets for patients to choose from in clinical settings.

## 6 Conclusion

Since ferroptosis was identified in 2012, researchers have expressed a burgeoning interest in understanding its association with various ophthalmic conditions. This manuscript delves into the regulatory mechanisms of ferroptosis and their potential impacts on eye diseases. However, numerous aspects of how ferroptosis is implicated in the regulation of ophthalmic diseases remain largely unknown. Further research is essential to explore the mechanisms and roles of ferroptosis in conditions such as corneal injury, glaucoma, cataract, age-related macular degeneration, diabetic retinopathy, retinal detachment, and retinoblastoma.

The role of cell type and sensitivity in ferroptosis is intricate and tissue-specific. In the context of ocular diseases, this implies that ocular cells may be more prone to ferroptosis, which deviates from its manifestation in other tissues such as tumor cells and neurons. For instance, the retina, the light-sensitive tissue of the eye, contains a large number of neurons and glial cells. In conditions like AMD and certain types of retinal degenerative diseases, retinal cells are more susceptible to the effects of ferroptosis. Ferroptosis drives the progression of these diseases by affecting these cells’ redox balance.

Due to its high energy consumption and highly oxidative physiological characteristics, the retina forms an environment particularly susceptible to oxidative stress. Photoreceptor cells require a substantial amount of energy to maintain the transmission of visual signals. Mitochondria are extremely active in these cells, generating energy while also producing reactive oxygen species (ROS), which heighten oxidative stress.

Moreover, the eyes are directly exposed to light, especially high-energy visible light and ultraviolet rays. Such exposure can promote the generation of ROS and cause oxidative damage to retinal cells. Given that ferroptosis is a form of cell death closely linked to oxidative stress, its significance is even more pronounced in the eyes.

The focus of future research on ferroptosis in ophthalmic diseases involves uncovering its molecular mechanisms, including the regulation of iron ion metabolism and lipid peroxidation. Understanding these mechanisms is critical for identifying key factors in the development of ophthalmic diseases. This knowledge can aid in discovering new therapeutic targets and biomarkers, thereby enabling early diagnosis and monitoring of disease progression.

As our understanding of the molecular mechanisms of ferroptosis deepens, researchers will endeavor to develop new drugs that can target these pathways. These include small molecule inhibitors and inducers, as well as exploring the application of gene editing technology and stem cell therapy in the treatment of ophthalmic diseases. These strategies may offer new approaches to treatment, particularly when conventional treatments prove ineffective.

Furthermore, the advancement of non-invasive diagnostic technology and interdisciplinary collaboration will be pivotal to advancing research on ferroptosis. Non-invasive techniques can empower doctors to intervene at an early stage of the disease, while interdisciplinary collaboration can harness the expertise of professionals from diverse fields to collectively address complex issues in the treatment of ophthalmic diseases. This collaboration can optimize treatment strategies and ultimately deliver more effective treatment plans to patients.

The current manuscript does have certain limitations. For instance, our exploration of ophthalmic diseases is restricted to eight specific types: corneal injury, cataract, glaucoma, AMD, DR, RP, RB, and RD. This does not encompass all ophthalmic diseases potentially related to ferroptosis, such as Graves’ ophthalmopathy (288) and myopia (289). Our literature search was confined to English-language studies, thus excluding research in other languages, including Chinese. The review may inevitably incorporate lower-quality studies, potentially impacting the reliability of the conclusions. There may also be publication bias in the conclusions, as studies with negative or non-significant results may not have been published, possibly skewing the review towards supporting the role of ferroptosis in ophthalmic diseases. Given these limitations, future research should address a broader range of diseases, adopt more rigorous study designs, and conduct more comprehensive investigation into mechanisms.

In summary, ferroptosis is considered a significant form of cell death in ophthalmology. A more comprehensive understanding of ferroptosis may reveal new targets for the diagnosis and treatment of ophthalmic diseases, offering promising avenues for future therapeutic advancements.

## Author contributions

YY: Writing – original draft. YL: Writing – original draft. ZH: Writing – original draft, Writing – review & editing. BW: Writing – original draft. WZ: Writing – review & editing. LW: Writing – review & editing.
